# Engineered interleukin-6-derived cytokines recruit artificial receptor complexes and disclose CNTF signaling *via* the OSMR

**DOI:** 10.1016/j.jbc.2024.107251

**Published:** 2024-04-01

**Authors:** Puyan Rafii, Patricia Rodrigues Cruz, Julia Ettich, Christiane Seibel, Giacomo Padrini, Christoph Wittich, Alexander Lang, Patrick Petzsch, Karl Köhrer, Jens M. Moll, Doreen M. Floss, Jürgen Scheller

**Affiliations:** 1 Institute of Biochemistry and Molecular Biology II, Medical Faculty, Heinrich-Heine-University, Düsseldorf, Germany; 2 Division of Cardiology, Pulmonology, and Vascular Medicine, Cardiovascular Research Laboratory, Medical Faculty, University Hospital Düsseldorf, Düsseldorf, Germany; 3 Biological and Medical Research Center (BMFZ), Medical Faculty, Heinrich-Heine-University, Duesseldorf, Germany

**Keywords:** gp130, LIFR, OSMR, IL-6, LIF, CNTF, cytokine, interleukin, signaling, receptor

## Abstract

Ciliary neurotrophic factor (CNTF) activates cells *via* the non-signaling α-receptor CNTF receptor (CNTFR) and the two signaling β-receptors glycoprotein 130 (gp130) and leukemia inhibitory factor receptor (LIFR). The CNTF derivate, Axokine, was protective against obesity and insulin resistance, but clinical development was halted by the emergence of CNTF antibodies. The chimeric cytokine IC7 used the framework of interleukin (IL-)6 with the LIFR-binding site from CNTF to activate cells *via* IL-6R:gp130:LIFR complexes. Similar to CNTF/Axokine, IC7 protected mice from obesity and insulin resistance. Here, we developed CNTF-independent chimeras that specifically target the IL-6R:gp130:LIFR complex. In GIL-6 and GIO-6, we transferred the LIFR binding site from LIF or OSM to IL-6, respectively. While GIO-6 signals *via* gp130:IL-6R:LIFR and gp130:IL-6R:OSMR complexes, GIL-6 selectively activates the IL-6R:gp130:LIFR receptor complex. By re-evaluation of IC7 and CNTF, we discovered the Oncostatin M receptor (OSMR) as an alternative non-canonical high-affinity receptor leading to IL-6R:OSMR:gp130 and CNTFR:OSMR:gp130 receptor complexes, respectively. The discovery of OSMR as an alternative high-affinity receptor for IC7 and CNTF designates GIL-6 as the first truly selective IL-6R:gp130:LIFR cytokine, whereas GIO-6 is a CNTF-free alternative for IC7.

In humans, the interleukin (IL)-6 family comprises the nine cytokines IL-6 (1), IL-11 (2), IL-27 (IL-30) (3), IL-31 (4), leukemia inhibitory factor (LIF) (5), oncostatin M (OSM) (4), ciliary neurotrophic factor (CNTF), cardiotrophin-1 (CT-1) (6), cardiotrophin-like cytokine factor 1 (CLCF1, previously named cardiotrophin-like cytokine (CLC)) (7). Apart from IL-31, all IL-6-type cytokines induce signal transduction *via* the common β-receptor gp130 (8, 9), which leads to the activation of signaling cascades including the JAK/STAT, Ras/Raf/MAP kinase and phosphatidylinositol 3-kinase pathways (10). Whereas IL-6 and IL-11 signal *via* gp130 homodimers, the other cytokines signal *via* gp130 heterodimers, exemplified by CNTF and LIF which recruit a heterodimer of gp130 and the LIF receptor (LIFR) (11, 12, 13) and human OSM which recruits either human gp130:OSMR or gp130:LIFR heterodimeric receptor complexes (14, 15). All IL-6-type cytokines have two β-receptor binding sites. IL-6, IL-11, CNTF, CLCF1, and IL-27p28 have an additional binding site for their initial interaction with the respective non-signaling α-receptor IL-6R, IL-11R, CNTFR, or EBI3 (11, 12, 16, 17), which is mandatory for β-receptor complex formation and initiation of signal transduction. The primary α-receptor binding site is therefore defined as site 1, whereas secondary β-receptor binding sites are named site 2 and site 3 (11, 12, 13, 18). Receptor recognition sites of IL-6-type cytokines have evolved with high sequence variability but with high structure homology. We and others have shown that at least site 3 is principally interchangeable resulting in the chimeric cytokines (cytokimera) IC7 and GIL-11. In IC7, site 3 from CNTF was transferred to IL-6 and the chimeric cytokine activated the non-natural receptor complex consisting of IL-6R:gp130:LIFR (19), whereas the transfer of site 3 from LIF to IL-11 resulting in GIL-11 led to the assembly of the non-natural receptor complex IL-11R:gp130:LIFR (20).

While GIL-11 accelerated liver regeneration after partial hepatectomy in mice (20), IC7 improved glucose tolerance and reduced hyperglycemia, thereby preventing weight gain and liver steatosis in mice (21). Other cytokines of the IL-6 cytokine family also conferred protective effects on obesity and insulin resistance, including the IC7 source cytokines IL-6 (22) and CNTF (23). Clinical development of the optimized CNTF-variant Axokine for weight control was, however, discontinued because about 70% of the probands in the phase III clinical trial developed neutralizing antibodies against the therapeutic agent (24). Since CNTF lacks a signal peptide, it either becomes available after cellular damage or through an unknown release mechanism (25). We assume that the sparse serum availability of CNTF might contribute to the fast development of auto-antibodies. Further assessment is required to see if repetitive injections of CNTF-derived chimeric cytokines such as IC7 will also provoke the development of auto-antibodies. Additionally, the major pro-inflammatory effects of IL-6 make its use as a means of weight control disadvantageous. One point worthy of note, however, is that the therapeutic application of IC7 in non-human primates did not provoke inflammatory conditions (21).

We developed CNTF-free cytokimera, which also targets IL-6R:gp130:LIFR complexes. These new cytokimera, GIL-6 and GIO-6, use IL-6 as a backbone and site 3 from LIF and OSM, respectively. They target the receptor complexes IL-6R:gp130:LIFR (GIL-6), and IL-6R:gp130:LIFR plus IL-6R:gp130:OSMR (GIO-6). During validation of the specifications of IC7, the alternative high-affinity non-natural receptor complex IL-6R:gp130:OSMR was assigned as an alternative receptor complex for IC7. This led to the identification of the alternative high-affinity natural receptor complex CNTFR:gp130:OSMR for CNTF.

## Results

### Structural design of the cytokimera GIL-6 and GIO-6

**Figure 1:**
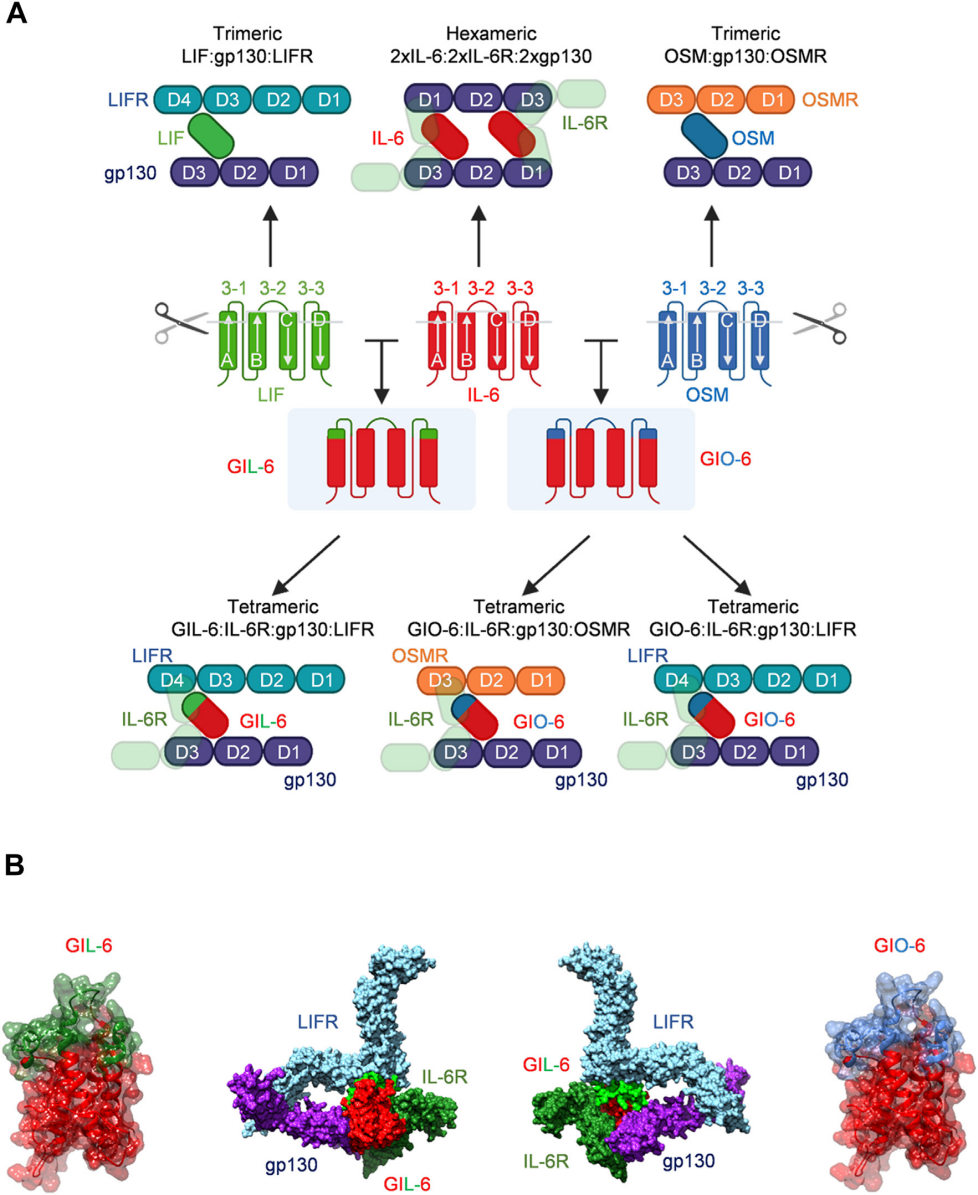
**Design of the cytokimera GIL-6 and GIO-6.***A*, schematic illustration of LIF within the trimeric LIF:gp130:LIFR complex, IL-6 in the hexameric 2xIL-6:2xIL-6R:2xgp130 complex, and OSM in the trimeric OSM:gp130:OSMR complex. IL-6 (*red*) was used as a scaffold for the generation of the cytokimera. Site 3 of IL-6 comprising of site 3-1, 3-2, and 3-3 was exchanged by site 3 of either LIF (*green*) leading to GIL-6 or by site 3 of OSM (*blue*) resulting in GIO-6. GIL-6 initially binds to D2/D3 of IL-6R *via* site 1. Subsequently, GIL-6 binds to D2/D3 of gp130 *via* site 2 of the IL-6 portion before GIL-6 binds to D3/D4 of the LIFR *via* site 3 of the LIF portion. GIL-6 forms a tetrameric GIL-6:IL-6R:gp130:LIFR complex. GIO-6 binds either to D2/D3 of the OSMR or to D3/D4 of the LIFR *via* site 3 of OSM, resulting in the tetrameric GIO-6:IL-6R:gp130:OSMR or GIO-6:IL-6R:gp130:LIFR complexes. *B*, superposed structure in ribbon and surface view of IL-6 (*red*) (1ALU) including the exchanged regions by LIF (*green*) or OSM (*blue*) for GIL-6 *left* and GIO-6 *right*, respectively. GIL-6 modelled in complex of the tetrameric GIL-6:IL-6R:gp130:LIFR complex (PDB 1ALU; 2Q7N; 1P9M) in surface view.

Using structure-based modeling with IL-6 as a backbone, we designed the two novel cytokimera GIL-6, and GIO-6 by replacing site 3 with the site 3 sections of LIF and OSM, respectively (Fig. 1*A*). The composite site 3 mediates binding of IL-6 to gp130, of LIF to LIFR and of OSM to LIFR or OSMR (13, 18) and consists of amino acid residues of the C-terminal α-helix and the N-terminal loop connecting helices A and B for site 3-1, the BC loop for site 3-2 and the C-terminal loop connecting helices C and D plus the N-terminal part of helix D for site 3-3 (11, 12). Human IL-6 consists of 212 amino acids including the signal peptide. Its site 3 consists of the amino acids R68-N88 (site 3-1), L129-R141 (site 3-2), and L179-R196 (site 3-3). LIF consists of 202 amino acids with site 3 ranging from amino acids F63-V86(site 3-1), I119-N138 (site 3-2), and D171-G189 (site 3-3). OSM has 221 amino acids with site 3 comprising the amino acids L56-P78 (site 3-1), L113-Q137 (site 3-2), and P176-H196 (site 3-3) (Figs. 1*B* and S1). The resulting cytokimera were larger than the natural cytokines with 240 amino acids for GIL-6, and 229 amino acids for GIO-6. *In silico* modeling using structure-based alignments suggested that the transfer of the complete site 3 should, however, not interfere with overall architecture (Fig. 1*B*). While IL-6 forms α-receptor-dependent hexameric receptor complex with 2xIL-6:2xIL-6R:2xgp130 (11, 26), the α-receptor-independent signaling complexes for LIF and OSM are trimeric, with LIF:gp130:LIFR, OSM:gp130:OSMR and OSM:gp130:LIFR (Fig. 1*A*) (13, 18, 27). The cytokimera GIL-6 and GIO-6 are designed to specifically form α-receptor-dependent tetrameric complexes consisting of GIL-6:IL-6R:gp130:LIFR, GIO-6:IL-6R:gp130:LIFR and GIO-6:IL-6R:gp130:LIFR (11, 13, 28, 29, 30, 31, 32, 33) (Fig. 1*A*).

### The cytokimera GIL-6 and GIL-6 promote signal transduction and cellular proliferation *via* non-natural cytokine receptor complexes

**Figure 2:**
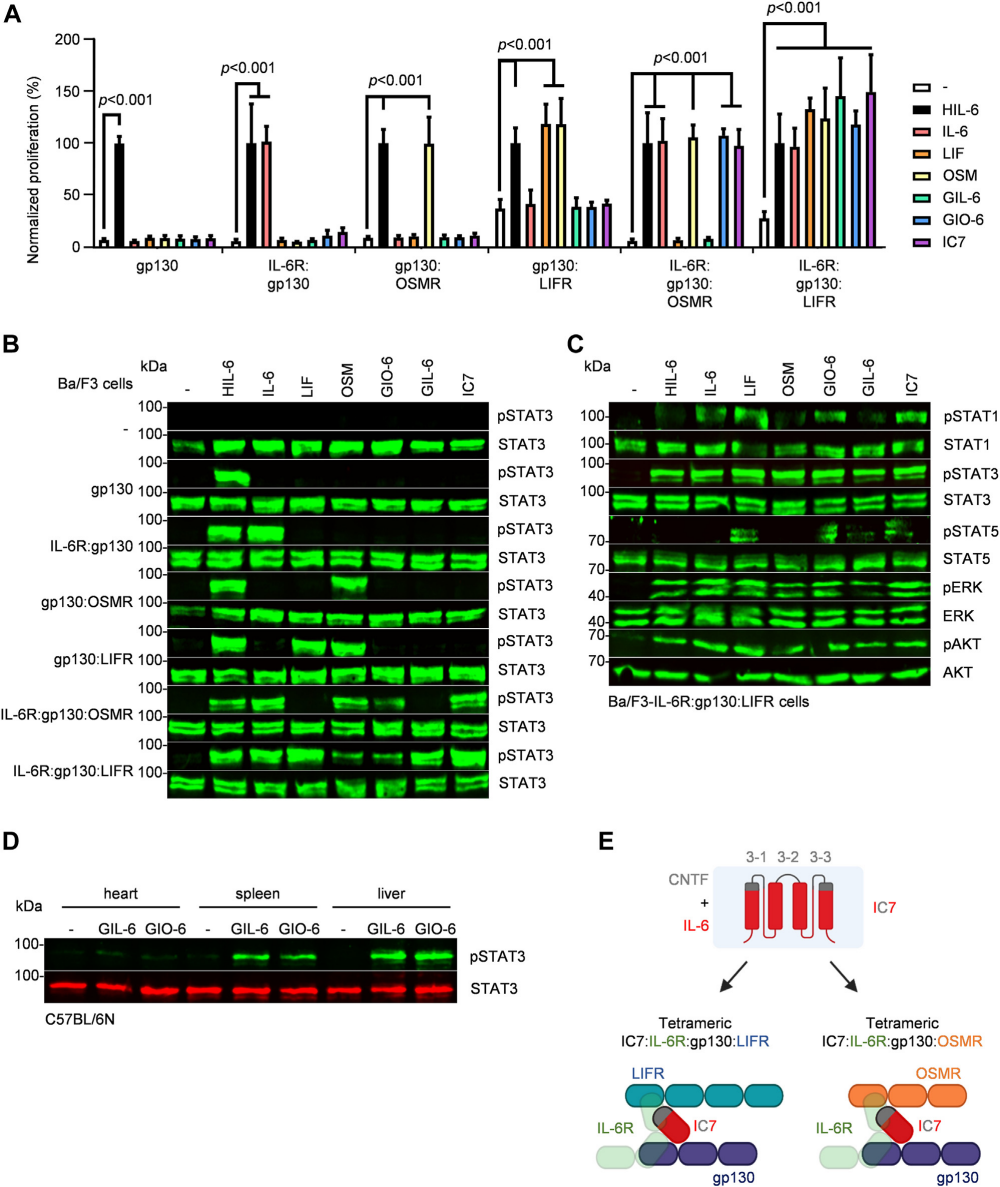
**GIL-6 induces JAK/STAT signaling and cellular proliferation *via* non-natural cytokine receptor complexes.***A*, proliferation of Ba/F3-gp130, Ba/F3-IL-6R:gp130, Ba/F3-gp130:OSMR, Ba/F3-gp130:LIFR, Ba/F3-IL-6R:gp130:OSMR, Ba/F3-IL-6R:gp130:OSMR, Ba/F3-IL-6R:gp130:LIFR cells without cytokine (−), with 100 ng/ml HIL-6, 10 ng/ml IL-6, 10 ng/ml LIF, 10 ng/ml OSM, 100 ng/ml GIL-6, 100 ng/ml GIO-6 and 100 ng/ml IC7. Data represents mean±S.D. of three independent experiments. For statistics the treated groups were compared with the untreated group by two-way ANOVA including Dunnet’s test for correction in multiple comparison. *B*, STAT3 activation in Ba/F3, Ba/F3-gp130, Ba/F3-IL-6R:gp130, Ba/F3-gp130:OSMR, Ba/F3-gp130:LIFR, Ba/F3-IL-6R:gp130:OSMR, Ba/F3-IL-6R:gp130:LIFR cells without cytokine (−) and after stimulation with 100 ng/ml HIL-6, 10 ng/ml IL-6, 10 ng/ml LIF, 10 ng/ml OSM, 100 ng/ml GIL-6, 100 ng/ml GIO-6 and 100 ng/ml IC7 for 20 min. *C*, STAT1, STAT3, STAT5, ERK, and Akt activation in Ba/F3-IL-6R:gp130:LIFR cells with the same conditions as for the STAT3 activation. *D*, STAT3 activation in heart, liver, and spleen after injection of 20 μg GIL-6 or GIO-6. Mice were sacrificed 30 min after intraperitoneal cytokine injection. Equal amounts of proteins (50 μg/lane) were analyzed *via* specific antibodies detecting phospho-STAT3 and STAT3. Western blot data shows one representative experiment out of three. *E*, schematic illustration of IC7. Site 3 of IL-6 (*red*) comprising site 3-1, 3-2, and 3-3 was exchanged by site III of CNTF (*grey*) resulting in IC7. Consequently, IC7 forms tetrameric IC7:IL-6R:gp130:LIFR or IC7:IL-6R:gp130:OSMR complexes.

IL-3 is required to induce the proliferation of the murine pre-B cell line Ba/F3 (34). After the introduction of the coding cDNA for human gp130 and additional receptors of the family, including IL-6R, IL-11R, OSMR, LIFR, and CNTFR (Fig. S2), the proliferation of Ba/F3 cells became responsive to the respective IL-6-type cytokine receptor combination (Fig. 2*A*). GIO-6 induced proliferation of cells expressing IL-6R:gp130:LIFR as well as IL-6R:gp130:OSMR, whereas GIL-6 exclusively induced proliferation of Ba/F3-IL-6R:gp130:LIFR cells (Fig. 2*A*). Hyper IL-6 (IL-6:soluble IL-6R fusion protein), IL-6, LIF, OSM, and the cytokimera IC7 (21) were used as controls to verify receptor specificity of the Ba/F3 cell repertoire. As expected, Hyper IL-6 induced proliferation of all cell lines because of the general expression of gp130 (35). After the addition of IL-6R, the derivative cell lines proliferated with IL-6. LIFR:gp130-expressing cells proliferated with LIF, and OSM, while OSM also induced the proliferation of OSMR:gp130-expressing cells. As previously described, IC7 induced the proliferation of Ba/F3-IL-6R:gp130:LIFR cells (21) but also of Ba/F3-IL-6R:gp130:OSMR cells (Fig. 2*A*). Next, we analyzed the STAT3 phosphorylation induced by cytokimera GIL-6, GIO-6, and IC7 and the cytokines HIL-6, IL-6, LIF, and OSM in the Ba/F3 cell collection. Importantly, the STAT3 phosphorylation observed in Ba/F3 cells (Fig. 2*B*) after cytokine stimulation mirrors the data obtained from proliferation assays (Fig. 2*A*). STAT3 phosphorylation was observed in Ba/F3 cells expressing gp130 after stimulation with HIL-6; IL-6R:gp130 with IL-6; gp130:LIFR with LIF and gp130:LIFR or gp130:OSMR with OSM. IC7 and GIO-6 induced STAT3 phosphorylation in IL-6R:gp130:LIFR and IL-6R:gp130:OSMR expressing Ba/F3 cells, whereas GIL-6 induced STAT3 phosphorylation selectively in Ba/F3-IL-6R:gp130:LIFR cells (Fig. 2*B*). To further characterize the primary signaling pathways, Ba/F3-IL-6R:gp130:LIFR cells were used as every cytokine and cytokimera tested had an effect on this cell line. As expected, all cytokines and cytokimera induced STAT3, ERK, and Akt phosphorylation. Strong STAT1 phosphorylation was visible for IL-6, LIF, GIO-6, and IC7. HIL-6, OSM, and GIL-6 also induced STAT1 phosphorylation, albeit to a lesser extent. A weak STAT5 phosphorylation was seen for LIF, GIO-6, GIL-6, and IC7 signaling. This indicates that STAT1 and STAT5 phosphorylation is LIFR-mediated, whilst taking place solely through gp130 in Hyper IL-6 and IL-6 signal transduction (Fig. 2*C*) (36).

Human IL-6, LIF, CNTF, OSM, and IC7 are biologically active in mice (15, 19, 21, 37, 38, 39, 40), which was also found for GIL-6 and GIO-6. The intraperitoneal injection of 10 μg GIL-6 and GIO-6 into wild-type C57BL/6N mice induced STAT3 phosphorylation in the liver, whereas only weak STAT3 phosphorylation was observed in the spleen whereas no signal was detected in the heart (Fig. 2*D*).

**Figure 3:**
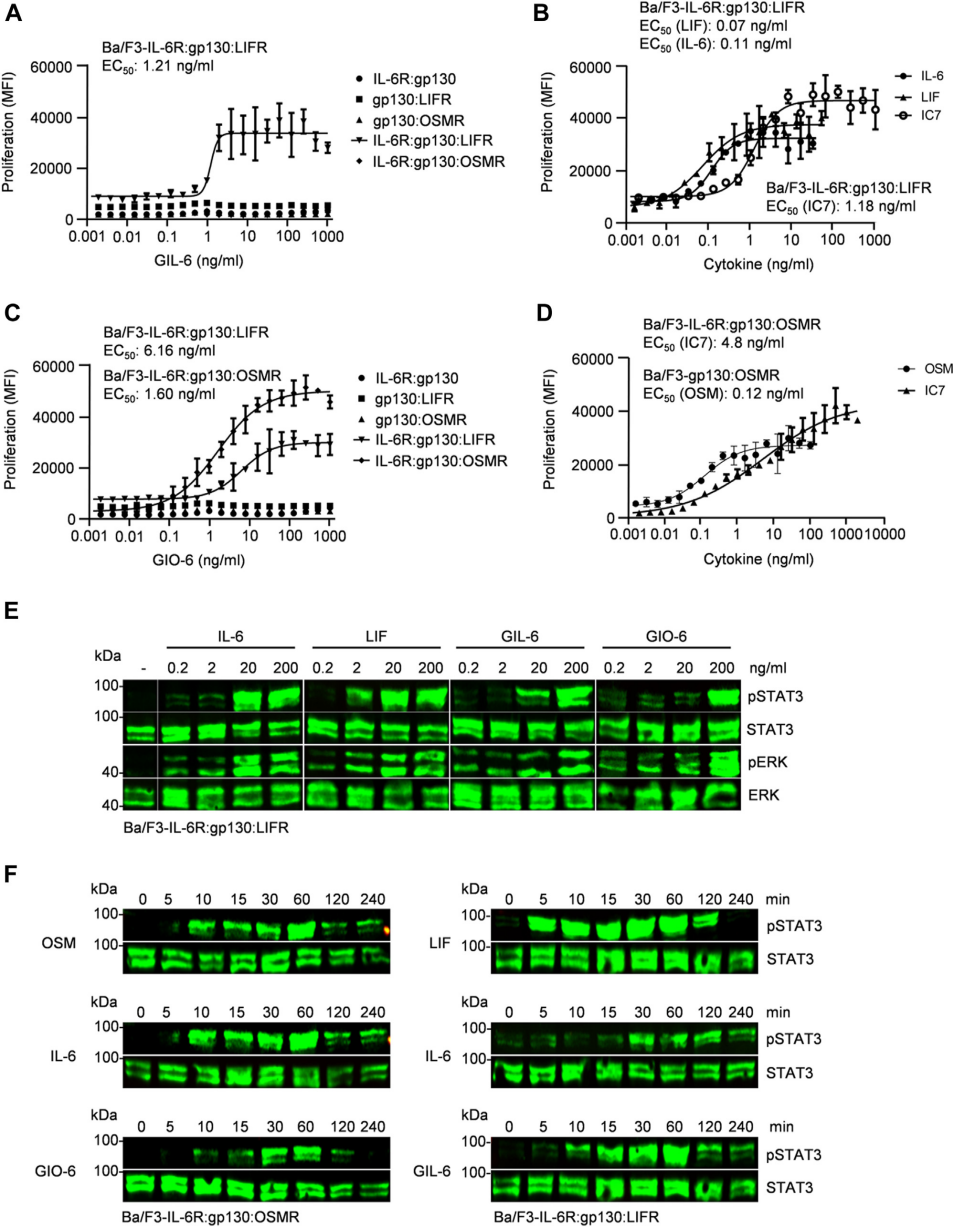
**Biological activity of cytokimeras GIL-6 and GIO-6 is comparable to natural cytokines.***A*, proliferation of Ba/F3-IL-6R:gp130, Ba/F3-gp130:LIFR, Ba/F3-gp130:OSMR, Ba/F3-IL-6R:gp130:LIFR, Ba/F3-IL-6R:gp130:OSMR cells in the presence and absence of increasing concentrations of GIL-6 (0.002–1000 ng/ml). The EC_50_ values were calculated by fitting a non-linear regression curve. One representative experiment out of three is shown. *B*, proliferation of Ba/F3-IL-6R:gp130:LIFR cells in the presence and absence of increasing concentrations LIF (0.002–50 ng/ml), IL-6 (0.002–30 ng/ml) or IC7 (0.002–1000 ng/ml). One representative experiment out of three is shown. *C*, proliferation of Ba/F3-IL-6R:gp130, Ba/F3-gp130:LIFR, Ba/F3-gp130:OSMR, Ba/F3-IL-6R:gp130:LIFR, Ba/F3-IL-6R:gp130:OSMR cells in the presence and absence of increasing concentrations of GIO-6 (0.002–1000 ng/ml). One representative experiment out of three is shown. *D*, proliferation of Ba/F3-IL-6R:gp130:OSMR cells in the presence and absence of increasing concentrations of IC7 (0.002–2000 ng/ml) and proliferation of Ba/F3-gp130:OSMR cells in the presence of OSM (0.001–100 ng/ml). One representative experiment out of three is shown. *E*, STAT3 and ERK activation in Ba/F3-IL-6R:gp130:LIFR cells without cytokine (−) and after stimulation with increasing amounts of IL-6, LIF, GIL-6 or GIO-6 (0.2, 2, 20, 200 ng/ml) for 20 min. Equal amounts of proteins (50 μg/lane) were analyzed *via* specific antibodies detecting phospho-STAT3, STAT3, phospho-ERK and ERK. Western blot data shows one representative experiment out of three. *F*, time-dependent STAT3 activation of Ba/F3-IL-6R:gp130:OSMR cells with OSM (10 ng/ml), IL-6 (10 ng/ml) or GIO-6 (100 ng/ml) and Ba/F3-IL-6R:gp130:LIFR cells with LIF (10 ng/ml), IL-6 (10 ng/ml) or GIL-6 (100 ng/ml) for the indicated time points. Equal amounts of proteins (50 μg/lane) were analyzed *via* specific antibodies detecting phospho-STAT3 and STAT3.

In Ba/F3-IL-6R:gp130:LIFR cells, an EC_50_ of 1.21 ng/ml was determined for GIL-6, which is above the values of commercially purchased cytokines IL-6 (EC_50_: 0.11 ng/ml), and LIF (EC_50_: 0.08 ng/ml) but in the same range as IC7 (EC_50_: 1.11 ng/ml) (Fig. 3, *A* and *B*). GIO-6 efficiently induced proliferation of Ba/F3 cells expressing IL-6R:gp130:OSMR with an EC_50_ of 1.60 ng/ml and of Ba/F3-IL-6R:gp130:LIFR with an EC_50_ of 6.16 ng/ml, thus showing a lower efficiency in comparison to IL-6, OSM and LIF (EC_50_: 0.11, 0.12 ng/ml and 0.08 ng/ml, respectively) but also in the range of IC7 (EC_50_: 1.21 ng/ml for IL-6R:gp130:LIFR and 4.8 ng/ml for IL-6R:gp130:OSMR) (Fig. 3, *A*, *C* and *D*). Next, STAT3 and ERK phosphorylation were analyzed in Ba/F3-IL-6R:gp130:LIFR cells after stimulation with increasing concentrations of IL-6, LIF, GIL-6, and GIO-6 (0.2, 2, 20, and 200 ng/ml). Western blotting showed that 20 ng/ml GIL-6 and IL-6 were sufficient to induce STAT3 and ERK phosphorylation; whereas 2 ng/ml LIF and 200 ng/ml GIO-6 were needed for sustained signaling (Fig. 3*E*). In order to determine the time response of signal transduction, Ba/F3-IL-6R:gp130:OSMR and Ba/F3-IL-6R:gp130:LIFR cells were stimulated for up to 240 min with LIF, OSM, IL-6, GIO-6, and GIL-6. As expected, STAT3 phosphorylation was typically induced as early as 5 to 10 min after cytokine addition and decreased after 120 to 240 min (Fig. 3*F*), which is due to negative feedback by SOCS3 (41).

Taken together, our data demonstrate that GIL-6 and GIO-6 are biologically active on cells expressing IL-6R:gp130:LIFR and IL-6R:gp130:OSMR, respectively. The activity of GIL-6 and GIO-6 is comparable to that of IC7 but weaker than the activity of the natural cytokines IL-6, LIF, and OSM. Both GIO-6 and IC7 showed cross-reactivity for LIFR and OSMR. Of note, IC7 also induced signaling *via* IL-6R:gp130:OSMR complexes (Fig. 2*E*). Our development and the re-evaluation of IC7 make GIL-6 the first IL-6R:gp130:LIFR-selective chimeric cytokine.

### GIL-6, GIO-6, and IC7 transcriptome profiles are distinct from those of natural cytokines

**Figure 4:**
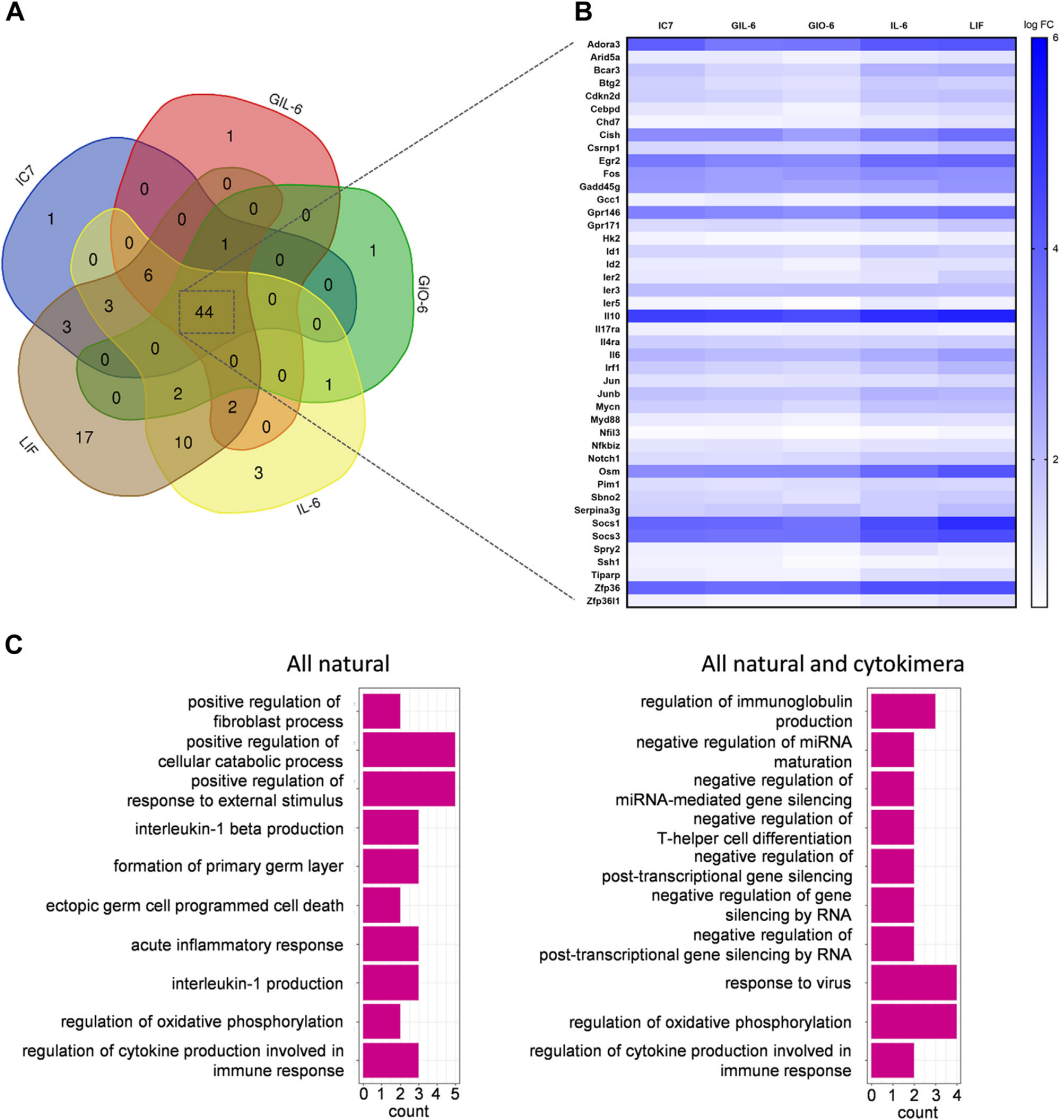
**Transcriptome profiles of GIL-6, GIO-6, and IC7 differ from natural cytokines.***A*, venn diagram shows an overlap of genes that are overexpressed following natural or synthetic cytokine stimulation of Ba/F3 cells expressing gp130, LIFR, and IL-6R. Filter: adjusted *p* < 0.05 including false discovery rate correction; |FC| >=1.5. *B*, heat map shows genes that are significantly increased by IC7, GIL-6, GIO-6, IL-6, or LIF *versus* untreated Ba/F3 cells expressing gp130, LIFR, and IL-6R. Scale bar shows log fold change. Filter: adjusted *p* < 0.05 including false discovery rate correction; |FC| >=1.5. *C*, gene ontology analysis of common gene pathways that are activated following stimulation of Ba/F3 cells expressing gp130, LIFR, and IL-6R following stimulation with natural cytokines IL-6, LIF, and synthetic cytokines. Filter: adjusted *p* < 0.05 including false discovery rate correction; |FC| >=1.5. X-axis: number of genes involved in common gene pathway.

To compare the transcription profiles of GIL-6, GIO-6, and IC7 with LIF and IL-6, Ba/F3-IL-6R:gp130:LIFR cells were stimulated with a 100fold EC_50_ concentration excess to achieve maximal induction of signal transduction. Among the genes showing at least a 1.5-fold increase in transcription, 44 were induced by the natural and chimeric cytokines (Fig. 4*A*, Table S1*A*, Figs. S3–S7). GIO-6 led to the weakest induction of transcription, whereas IC7 and GIL-6 led to a moderate and IL-6 and LIF to the strongest induction of gene expression (Fig. 4*B*). All cytokines induced typical IL-6-type cytokine target genes such as SOCS1 and SOCS3. Notably, Prickle1 is exclusively induced by IC7, Vmn1r47 solely by GIL-6, and Ifitm5 only by GIO-6 (Table S1, *A* and *B*). Gene ontology enrichment analysis revealed further differences: while genes induced by the natural cytokines are dominated by positive regulation of cellular catabolic processes and by responses to external stimuli, genes encoding proteins responsible for virus defense and leukocyte cell-cell adhesion are induced by both, natural and chimeric cytokines (Fig. 4*C*).

Taken together, the transcription profiles caused by the cytokimera differ from those of natural cytokines. Moreover, certain genes are exclusively activated by the chimeric cytokines.

### The cytokimera GIL-6 and GIO-6 are no effective *trans*-signaling inducers

**Figure 5:**
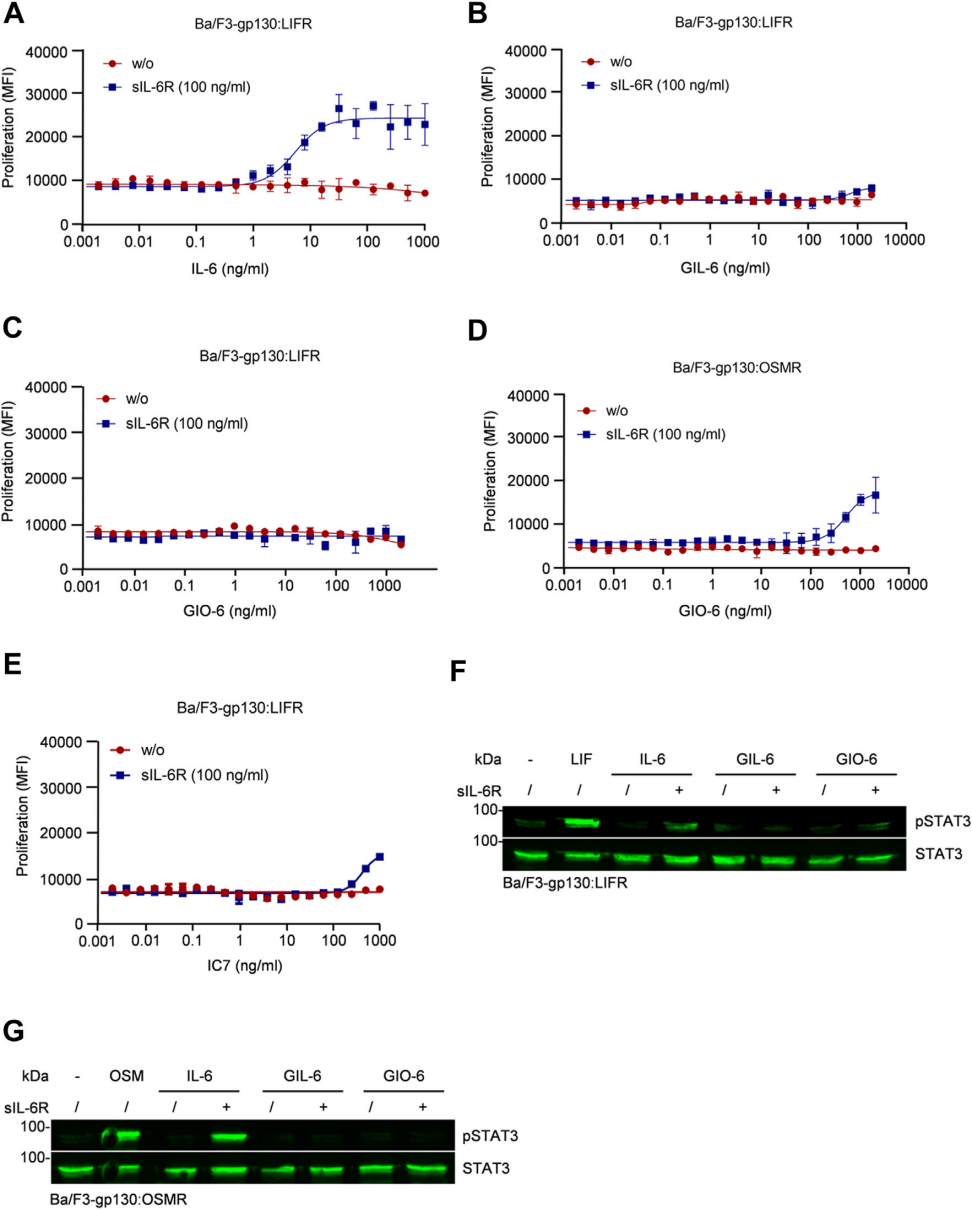
**Cytokimera GIL-6 and GIO-6 are poor inducers of *trans*-signaling.***A*, proliferation of Ba/F3-gp130:LIFR cells in the presence and absence of fixed concentrations of sIL-6R (0 or 100 ng/ml) and increasing concentrations of IL-6 (0.002–1000 ng/ml). One representative experiment out of three is shown. *B*, proliferation of Ba/F3-gp130:LIFR cells in the presence and absence of fixed concentrations of sIL-6R (0 or 100 ng/ml) and increasing concentrations of GIL-6 (0.002–2000 ng/ml). One representative experiment out of three is shown. *C*, proliferation of Ba/F3-gp130:LIFR cells in the presence and absence of fixed concentrations of sIL-6R (0 or 100 ng/ml) and increasing concentrations of GIO-6 (0.002–2000 ng/ml). One representative experiment out of three is shown. *D*, proliferation of Ba/F3-gp130:OSMR cells in the presence and absence of fixed concentrations of sIL-6R (0 or 100 ng/ml) and increasing concentrations of GIO-6 (0.002–2000 ng/ml). One representative experiment out of three is shown. *E*, proliferation of Ba/F3-gp130:LIFR cells in the presence and absence of fixed concentrations of sIL-6R (0 or 100 ng/ml) and increasing concentrations of IC7 (0.002–1000 ng/ml). One representative experiment out of three is shown. *F*, STAT3 activation in Ba/F3-gp130:LIFR cells without cytokine (−), 10 ng/ml LIF, 100 ng/ml IL-6, 100 ng/ml GIL-6, 100 ng/ml GIO-6 and either with 100 ng/ml sIL-6R (+) or without (/). Equal amounts of protein (50 μg/lane) were analyzed *via* specific antibodies detecting phospho-STAT3 and STAT3. Western blot data shows one representative experiment out of three. *G*, STAT3 activation in Ba/F3-gp130:OSMR cells without cytokine (−), 10 ng/ml OSM, 100 ng/ml IL-6, 100 ng/ml GIL-6, 100 ng/ml GIO-6 and either with 100 ng/ml sIL-6R (+) or without (/). Equal amounts of protein (50 μg/lane) were analyzed *via* specific antibodies detecting phospho-STAT3 and STAT3. Western blot data shows one representative experiment out of three.

In classic signaling, IL-6 interacts with, membrane-bound IL-6R to then induce the homodimerization of gp130. IL-6 can also bind to the soluble IL-6R prior to forming a complex with gp130, which is called *trans*-signaling (42, 43). As IL-6 *trans*-signaling is known to be a key mediator of chronic inflammatory diseases (42, 43), we investigated the cytokimeras’ capacity to promote *trans*-signaling. Ba/F3 cells expressing gp130:LIFR or gp130:OSMR were stimulated with increasing concentrations of GIL-6, GIO-6, and IL-6 (0.0005–2000 ng/ml) in the presence and absence of 100 ng/ml soluble IL-6R (sIL-6R). Whereas IL-6:sIL-6R complexes induced proliferation of Ba/F3-gp130-LIFR cells in a dose-dependent manner with an EC_50_ of 5.3 ng/ml, GIL-6 and GIO-6 were not able to induce proliferation of Ba/F3-gp130:LIFR at any concentration tested (Fig. 5, *A*–*C*). Only using concentrations above 500 ng/ml, GIO-6 and IC7 led to a weak proliferation of Ba/F3-gp130:OSMR and Ba/F3-gp130:LIFR cells, respectively (Fig. 5, *D* and *E*). In the proliferation assay performed, GIL-6 and GIO-6 (100 ng/ml) did not activate downstream STAT3 phosphorylation *via trans*-signaling using 100 ng/ml sIL-6R (Fig. 5, *F* and *G*). Therefore, we conclude that GIL-6, GIO-6, and IC7 are not effective in inducing *trans*-signaling.

### CNTF signals *via* the CNTFR:gp130:OSMR complex

**Figure 6:**
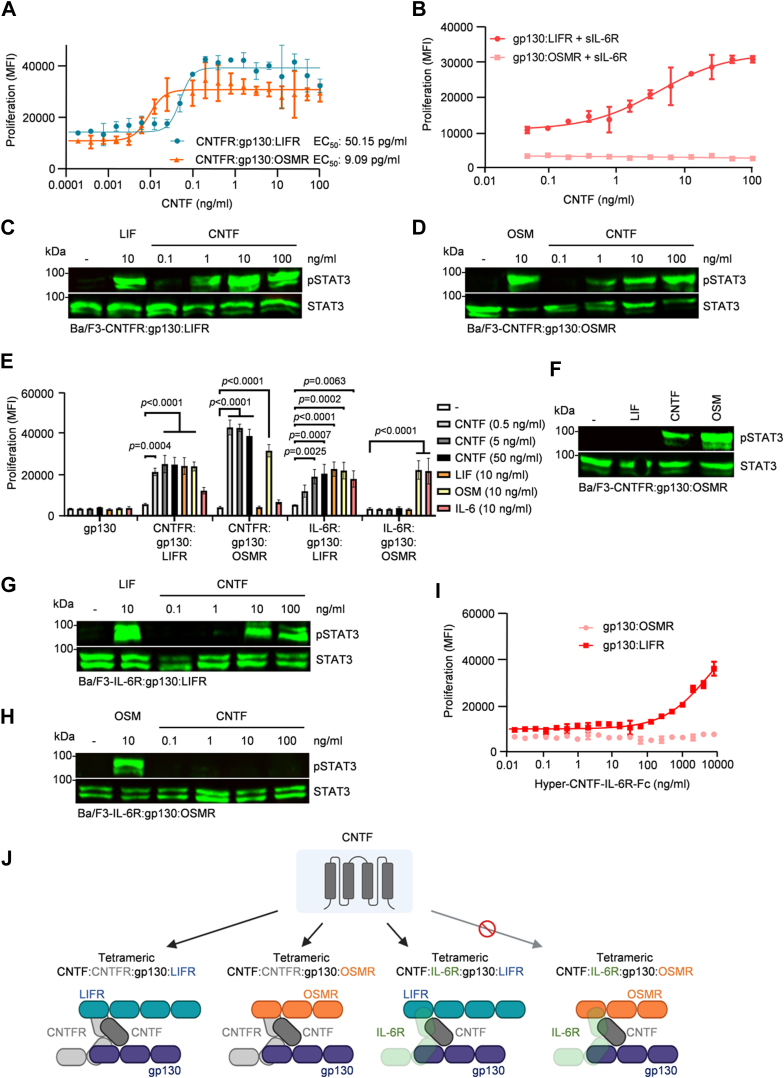
**CNTF signals *via* the alternative CNTFR:gp130:OSMR complex but not *via* IL-6R:gp130:OSMR.***A*, proliferation of Ba/F3-CNTFR:gp130:LIFR or Ba/F3-CNTFR:gp130:OSMR cells with increasing concentrations of CNTF (0.0002–100 ng/ml). One representative experiment out of four is shown. *B*, proliferation of Ba/F3-gp130:LIFR or Ba/F3-gp130:OSMR cells in the presence of fixed concentrations of sIL-6R (100 ng/ml) and increasing concentrations of CNTF (0.05–100 ng/ml). One representative experiment out of three is shown. *C* and *D*, CNTF dose-dependent STAT3 activation of Ba/F3-CNTFR:gp130:LIFR (*C*), Ba/F3-CNTFR:gp130:OSMR (*D*) cells without cytokine (−) or in the presence of 10 ng/ml LIF or OSM, or with 0.1, 1, 10 or 100 ng/ml CNTF for 20 min. Equal amounts of proteins (50 μg/lane) were analyzed *via* specific antibodies detecting phospho-STAT3 and STAT3. Western blot data shows one representative experiment out of three. *E*, proliferation of Ba/F3-gp130, Ba/F3-CNTFR:gp130:LIFR, Ba/F3-CNTFR:gp130:OSMR, Ba/F3-IL-6R:gp130:LIFR or Ba/F3-IL-6R:gp130:OSMR cells without cytokine (−) or in the presence of CNTF (0.5, 5 or 50 ng/ml), LIF (10 ng/ml), OSM (10 ng/ml) or IL-6 (10 ng/ml). Data represents mean ± S.D. of three independent experiments. For statistics, the treated groups were compared with the untreated group by two-way ANOVA including Dunnet’s test for correction in multiple comparisons. *F*, STAT3 activation Ba/F3-CNTFR:gp130:OSMR cells without cytokine (−), 10 ng/ml LIF, 10 ng/ml CNTF or 10 ng/ml OSM. Equal amounts of proteins (50 μg/lane) were analyzed *via* specific antibodies detecting phospho-STAT3 and STAT3. Western blot data shows one representative experiment out of three. *G* and *H*, CNTF dose-dependent STAT3 activation of Ba/F3-IL-6R:gp130:LIFR (*G*) or Ba/F3-IL-6R:gp130:OSMR (*H*) cells without cytokine (−) or in the presence of 10 ng/ml LIF or OSM, or with 0.1, 1, 10 or 100 ng/ml CNTF for 20 min. Equal amounts of proteins (50 μg/lane) were analyzed *via* specific antibodies detecting phospho-STAT3 and STAT3. Western blot data shows one representative experiment out of three. *I*, proliferation of Ba/F3-gp130:LIFR or Ba/F3-gp130:OSMR cells in the presence of increasing concentrations of Hyper-CNTF-IL-6R-Fc (0.02–8000 ng/ml). One representative experiment out of three is shown. *J*, schematic illustration of CNTF in tetrameric complexes consisting of CNTF:CNTFR:gp130:LIFR, CNTF:CNTFR:gp130:OSMR or CNTF:IL-6R:gp130:LIFR but not CNTF:IL-6R:gp130:OSMR.

Since we showed that IC7 signals *via* OSMR (Fig. 3*D*), we found that also CNTF signals *via* OSMR using Ba/F3-CNTFR:gp130:OSMR cells (Fig. 6*A*). Notably, the EC_50_ value for CNTF to induce proliferation of Ba/F3-CNTFR:gp130:OSMR (EC_50_: 9.09 pg/ml) is lower than the EC_50_ value of Ba/F3-CNTFR:gp130:LIFR cells (EC_50_: 50.15 pg/ml) (Fig. 6*A*). Western blotting revealed STAT3 phosphorylation in Ba/F3-CNTFR:gp130:OSMR cells stimulated with 10 ng/ml CNTF or OSM, but not when stimulated with LIF (Fig. 6*F*). As depicted in Figure 6, *C* and *D*, 1 ng/ml CNTF was sufficient to induce STAT3 phosphorylation in Ba/F3-CNTFR:gp130:LIFR and Ba/F3-CNTFR:gp130:OSMR cells. CNTF is also able to recruit IL-6R instead of CNTFR as an alternative low-affinity α-receptor in CNTF:IL-6R:gp130:LIFR complexes (44). Therefore, we tested if CNTF also signals *via* the IL-6R:gp130:OSMR receptor complex. Various Ba/F3 cell lines were stimulated with 0.5, 5, and 50 ng/ml CNTF, 10 ng/ml LIF, OSM, IL-6, or were left untreated. As expected, proliferation of Ba/F3-gp130 cells was not induced by these cytokines (Fig. 6*E*). Proliferation of Ba/F3-CNTFR:gp130:LIFR and Ba/F3-IL-6R:gp130:LIFR cells was induced by CNTF, confirming the IL-6R cross-talk of CNTF, whilst proliferation of Ba/F3-IL-6R:gp130:OSMR cells was not induced by CNTF (Fig. 6*E*). STAT3 phosphorylation in Ba/F3-IL-6R:gp130:LIFR and Ba/F3-IL-6R:gp130:OSMR cells was determined after stimulation with 0.1, 1, 10, and 100 ng/ml CNTF. Whereas a concentration of 10 ng/ml CNTF was sufficient to induce STAT3 phosphorylation in Ba/F3-IL-6R:gp130:LIFR cells, even the much higher concentration of 100 ng/ml CNTF failed to induce STAT3 phosphorylation in Ba/F3-IL-6R:gp130:OSMR cells (Fig. 6, *G* and *H*). CNTF *trans*-signaling *via* sIL-6R led to the proliferation of Ba/F3-gp130:LIFR cells but not of Ba/F3-gp130:OSMR cells (Fig. 6*B*). We were interested to see whether a chimeric hyper-cytokine made out of CNTF and sIL-6R fused to an Fc part of an IgG1 antibody (Hyper-CNTF-IL-6R-Fc) would also fail to signal *via* gp130:OSMR complexes, which the proliferation assay performed showed to be the case. Hyper-CNTF-IL-6R-Fc specifically induced the proliferation of Ba/F3 cells expressing gp130:LIFR but not of cells expressing gp130:OSMR (Fig. 6*I*). Overall, our findings show that CNTF can employ OSMR as an alternative receptor after binding to CNTFR but not after binding to IL-6R (Fig. 6*J*).

**Figure 7:**
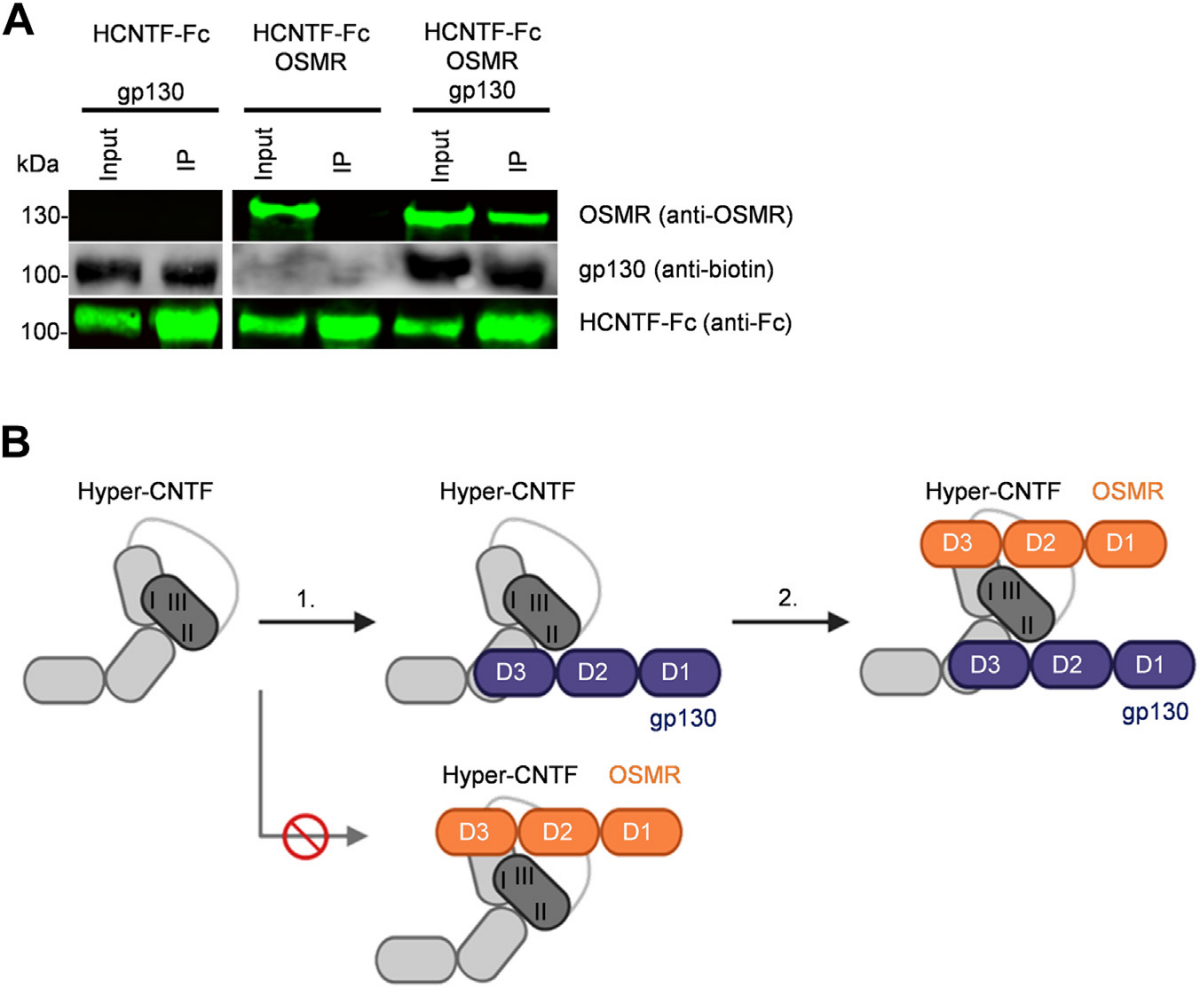
**CNTF recruits gp130 prior to OSMR.***A*, Western blotting of co-immunoprecipitation by using Protein A beads to precipitate recombinant Hyper-CNTF-Fc (2 μg), soluble OSMR (1 μg) in the presence or absence of biotinylated gp130 (1 μg). *B*, binding scheme of Hyper-CNTF. First Hyper-CNTF binds *via* site 2 to D2/D3 of gp130. The complex of Hyper-CNTF and gp130 binds hypothetically *via* site 3 to D2/D3 of OSMR. Hyper-CNTF is not able to bind the OSMR before recruiting gp130.

To investigate the binding order of CNTF to its receptors, Hyper-CNTF-Fc was precipitated by Protein A beads in the presence or absence of recombinant soluble OSMR or gp130. Hyper-CNTF-Fc is a fusion protein of CNTF and the soluble CNTFR (45) fused to an IgG_1_-derived Fc-part. OSM binds to gp130 *via* site 2 prior followed by the recruitment of the OSMR *via* site 3 (46). Therefore, we assumed that the binding of CNTF to the OSMR is also dependent on gp130. As shown in Figure 7*A*, OSMR was precipitated by Hyper-CNTF-Fc only in the presence of soluble gp130. In contrast, sgp130 was precipitated by Hyper-CNTF-Fc also in the absence of OSMR, demonstrating that CNTF needs to recruit gp130 prior to OSMR (Fig. 7*B*).

## Discussion

The rationale for the generation of chimeric cytokines such as the IC7 prototype is that the recruitment of non-natural receptor complexes results in a gain of cellular selectivity, thereby promoting beneficial outcomes and reducing negative side effects *in vivo* (47). Whereas the common β-receptor gp130 is ubiquitously expressed, expression of α-receptors and other β-receptors is restricted and therefore more cell-type specific, *e.g.* IL-6R is mainly found on immune cells and hepatocytes (48, 49) and confers a second layer of cellular specificity for IL-signaling (50). We designed, expressed and functionally characterized the two novel human chimeric designer cytokines GIL-6 and GIO-6 that signal *via* the non-natural receptor complexes IL-6R:gp130:LIFR and IL-6R:gp130:OSMR. The transfer of site 3 from an α-receptor-independent cytokine such as LIF to GIL-11 and GIL-6, or OSM to GIO-6 results in α-receptor-dependent chimeric cytokines, a result that was not entirely predictable. Our findings add to the evidence that although the original LIF/OSM-site 3 is α-receptor-independent, reformatting of LIF/OSM-site 3 into the IL-6 scaffold makes the site 3 of LIF and OSM α-receptor-dependent, suggesting that the interconnection mediated by α-helical shifts of site 1 with site 3 determines if a cytokine is α-receptor-dependent (51). Notably, the biological activity of GIL-6 and GIO-6 is comparable to IC7 and might be increased by the introduction of affinity-enhancing mutations in site 1 (52). Furthermore, the transcriptomic profiles of GIL-6 and GIO-6 were comparable but not identical with IC7, IL-6, LIF, or OSM. Some genes are exclusively induced by the synthetic cytokines, for example, Prickle1 and IFTM5. Prickle1 is crucial in early heart development (53, 54). Since Prickle1 is also known to regulate the differentiation of frontal bone osteoblast, our data might explain the contribution of IC7 to increasing bone density (21, 55). The interferon-induced transmembrane protein 5 (*IFTM5*) is selectively induced by GIO-6 and supports bone mineralization. Defects in IFTM5 signaling are associated with skeletal deformity in osteogenesis imperfecta patients (56, 57).

Revisiting IC7 led to the identification of OSMR as a second natural high-affinity receptor for CNTF. The expression of LIFR and OSMR is largely overlapping in many cell types including breast glandular cells, cholangiocytes, neurons, astrocytes, skeletal myocytes, smooth muscle cells, hepatocytes, and fibroblasts (58) (proteinatlas.org). Therefore, it is not surprising that OSMR was not previously described as an alternative receptor to the CNTF receptor. Heterologous expression of definite receptor chains in Ba/F3 cells proves to be a valid approach in identifying and validating receptor complexes of IL-6 type cytokines and cytokimera. CNTF plays an important protective role during the injury response of the nervous system (59, 60). Moreover, CNTF-deficient mice showed motor neuron degeneration, demonstrating its physiological role not only as an emergency factor but also in the maintenance of motor neurons (61). However, future studies must address the role of CNTF signaling *via* CNTFR:gp130:OSMR complexes. Since the application of CNTF prevented the degeneration of motor neurons after axotomy (62) and promoted survival of hippocampal neurons in culture (63), it would also be interesting to investigate if this effect is dependent on LIFR or OSMR signaling. Down the same lane, which receptor confers the beneficial effects of CNTF and IC7 on weight maintenance (19, 21, 64). The cytokimera GIL-6 differentiates between LIFR and OSMR signaling and is therefore promising therapeutic candidate protein.

In conclusion, our study showed the development of the two cytokimeras GIL-6 and GIO-6 with specific high-affinity activation of the non-natural receptor complexes consisting of IL-6R:gp130:LIFR and IL-6R:gp130:OSMR. Moreover, we showed that the previously described cytokine IC7 and also the natural cytokine CNTF use OSMR as an alternative β-receptor.

## Experimental procedures

### Cloning

The codon-optimized cDNA coding for GIL-6 and GIO-6 was ordered by BioCat GmbH (Heidelberg, Germany). The cDNA was then inserted into pcDNA3.1 expression vector including 5′ signal peptide for human IL-11R (Q14626, aa M1-A22) followed by sequences for myc tag (EQKLISEEDL) and the fragment encoding for the cytokimera a Gly4Ser linker and an Fc portion of an IgG1.

### Molecular modeling

Protein models were generated *via* the Phyre2 web portal. Complex models and structure-based sequence alignments were generated using UCSF Chimera version 1.17, developed by the Resource for Biocomputing, Visualization, and Informatics at the University of California, San Francisco, with support from NIH P41-GM103311.

### Cells, reagents, and recombinant proteins

The generation of Ba/F3-gp130 and Ba/F3-gp130:IL-6R cells was described elsewhere (65). The packaging cell line Phoenix-Eco was received from Ursula Klingmüller (DKFZ). All cells were grown at 37 °C with 5% CO_2_ in a water-saturated atmosphere in Dulbecco`s modified Eagle`s medium (DMEM) high-glucose (GIBCO, Life Technologies) with 10% fetal calf serum (GIBCO, Life Technologies) and 60 mg/l penicillin and 100 mg/l streptomycin (Genaxxon Bioscience GmbH). Murine Ba/F3-gp130 cells were obtained from Immunex and grown in the presence of HIL-6 0.2% (10 ng/ml) conditioned medium from a stable clone of CHO-K1 cells secreting HIL-6 in the supernatant (35). Expi-293F cells (ThermoFisher Scientific) were cultured in Expi293 expression medium without antibiotics until they reached a density of 3 to 5 × 10^6^ cells/ml in a 37 °C incubator with 8% CO_2_ on an orbital shaker at 125 rpm. Synthetic ligands were expressed and purified as described (66). Recombinant human OSM (catalog no.295-OM), LIF (catalog no. 7734-LF), and CNTF (catalog no. 257-NT) were purchased from R&D Systems (Minneapolis). Recombinant human IL-6 (catalog no.170-076-104) was purchased from Miltenyi Biotec GmbH (Bergisch Gladbach).

### Stimulation assay

Ba/F3 cell lines were washed three times with PBS to remove cytokines and starved in serum-free DMEM for 3 h. Cells were stimulated for 15 min (or as indicated) with purified protein (concentration as indicated), harvested, frozen in liquid nitrogen and then lysed. Cells were lysed for 45 min with buffer containing 10 mM Tris-HCl, pH 7.5, 150 mM NaCl, 0.5 mM MgCl_2_ and a cOmplete, EDTA-free protease inhibitor mixture tablet (Roche Diagnostics, Mannheim). Protein concentration was determined by a BCA protein assay (Thermo Fisher Scientific) according to the manufacturer`s instructions. Protein expression and pathway activation were then analyzed by Western blotting.

### Western blotting

50 μg total protein was loaded in each lane and separated by SDS-PAGE under reducing conditions and transferred to a nitrocellulose membrane (Amersham Protan; Cytiva; LC, United Kingdom; catalog no. 10600016). Blocking of the membrane was performed with blocking buffer (Intercept Blocking Buffer; LI-COR; catalog no. 927-60001) diluted 1:3 in TBS (10 mM Tris-HCl pH 7.6, 150 mM NaCl) for 1 h. Primary antibodies Phospho-STAT3 (Tyr-705; catalog no. 9145), STAT3 (catalog no. 9139), Erk1/2 (catalog no. 4696), Phospho-Erk1/2 (catalog no. 4370), Phospho-STAT1 (catalog no. 9167), STAT5 (catalog no. 94205), Phospho-STAT5 (catalog no. 9359), Akt (catalog no. 9272), Phospho-Akt (catalog no. 4060), myc (catalog no. 2278) (Cell Signaling Technology) were diluted 1:1000. Anti-OSMR (catalog no. BAF4389), LIFR (catalog no. BAF249) (R&D Systems) were diluted 1:2000. Anti-Fc (catalog no. 31423) (Thermo Scientific) was diluted 1:1000. All antibodies were diluted in blocking buffer containing 0.2% Tween-20 (Sigma-Aldrich; catalog no. P1379-1L) for at least 90 min at ambient temperature or overnight at 4 °C. Membranes were washed with TBS-T (0.1% Tween-20) and then incubated with secondary fluorophore-conjugated antibodies 1:10,000 (IRDye 800CW Donkey anti-Rabbit; catalog no. 926-32213 and IRDye 680RD Donkey anti-Mouse; catalog no. 926-68072, LI-COR; USA or with IRDye 800CW Donkey anti-Goat; catalog no. 926-32214) for 1 h. Signal detection was achieved using LI-COR (Odyssey; Model 2800). Secondary antibodies were detected simultaneously on different channels. Data analysis was conducted using Image Studio Lite 5.2. Liver, spleen, and heart tissue were lysed in lysis buffer (50 mM Tris HCl pH 7.5, 150 mM NaCl, 2 mM EDTA pH 8.0, 2 mM NaF, 1 mM Na_3_VO_4_, 1% NP-40, 1% Triton X-100, 1 cOmplete protease inhibitor cocktail tablet/50 ml). After lysis, the protein content was measured by BCA assay. 50 μg total protein amount was then loaded on each line followed by Immunoblotting. Antibodies used for blotting of lysed animal organs were as follows: anti-p-STAT3 (catalog no. 9145), anti-total-STAT3 (catalog no. 9139) (Cell Signaling Technology, USA). The detection of biotinylated gp130 (catalog no. AVI11067-050) was performed by using streptavidin-HRP (catalog no. 4800-30-06) diluted 1:300. HRP substrate was mixed 1:1 by using peroxide solution and luminol reagent (catalog no.WBKLS0500; EMD Millipore Corporation, Burlington) prior the chemiluminescence detection by the LI-COR Odyssey device.

### Cell viability assay

Ba/F3-gp130 cell lines were washed three times with PBS to remove cytokines from the medium. Cells with a density of 5 × 10^4^ cells/ml were suspended in DMEM containing 10% fetal calf serum, 60 mg/l penicillin, and 100 mg/ml streptomycin. Cells were cultured for 3 days in a volume of 100 μl with or without cytokines or inhibitors in the indicated concentrations. The CellTiter Blue Viability Assay (Promega) was used to determine the approximate number of viable cells by measuring the fluorescence (λ_excitation_560 nm/λ_emission_590 nm) using the Infinite M200 Pro plate reader (Tecan). After adding 20 μl/well of CellTiter Blue reagent (time point 0), fluorescence was measured after 60 min every 20 min for up to 2 h. For each condition of an experiment, 3 wells were measured. All values were normalized by subtracting time point 0 values from the final measurement. The EC_50_ values were calculated by fitting a nonlinear regression curve.

### Transduction of cells

Ba/F3-gp130 cell lines were retrovirally transduced with the pMOWS expression plasmids as described (34). Transduced cells were grown in DMEM medium as described above supplemented with 10 ng/ml HIL-6. Selection of transduced Ba/F3-gp130 cells was performed with puromycin (1.5 μg/ml) or hygromycin B (1 mg/ml) (Carl Roth, Karlsruhe, Germany) for at least 2 weeks. Afterward, the generated Ba/F3-gp130 cell lines were analyzed for receptor cell surface expression *via* flow cytometry.

### Cell surface detection of cytokine receptors *via* flow cytometry

Cell surface expression of stably transfected Ba/F3-gp130 cell lines was detected by specific antibodies. 5 × 10^5^ cells were washed in FACS buffer (PBS, 1% BSA) and then incubated in 50 μl of FACS buffer containing the indicated specific primary antibody (anti-LIFR, -OSMR or -CNTFR; 1:20; catalog no. BAF249 and BAF4389, and AF303-NA, R&D Systems). After incubation of at least 1 h at room temperature, cells were washed and resuspended in 50 μl of FACS buffer containing secondary antibody (NothernLights 493-conjugated anti-goat IgG 1:100) and incubated for 30 min at room temperature. Cells were washed and resuspended in 500 μl of FACS buffer and analyzed by flow cytometry (BD FACSCanto II flow cytometer using the FACSDiva software, BD Biosciences). Data analysis was conducted using FlowJo Version 10 (Tree Star Inc).

### 3′-RNA-Seq analysis and statistical analysis

Ba/F3-gp130-IL-6R:LIFR cells were stimulated with GIL-6, GIO-6, IC7, IL-6 or LIF for 40 min at 37 °C in a concentration of 100-fold EC_50_ that was previously determined. Each cytokine was analyzed separately. Four replicates were performed for every condition. mRNA was isolated with NucleoSpin RNA (Macherey-Nagel; cat. no. 740955.250) according to vendor’s manual. DNase digested total RNA samples used for 3′-RNA Seq analyses were quantified (Qubit RNA HS Assay, Thermo Fisher Scientific) and quality was measured by capillary electrophoresis using the Fragment Analyzer and the ‘Total RNA standard Sensitivity Assay’ (Agilent Technologies). All samples in this study showed very high-quality RNA Quality Numbers (RQN; mean = 10.0). The library preparation was performed according to the manufacturer’s protocol using the QuantSeq 3′ mRNA-Seq Library Prep Kit FWD from Lexogen. Input mount was 200 ng total RNA. Bead-purified libraries were normalized and finally sequenced on the NextSeq2000 system (Illumina Inc) with a read setup of SR 1 × 100 bp. The Illumina DRAGEN FASTQ Generation tool (version 3.8.4) was used to convert the bcl files to fastq files as well for adapter trimming and demultiplexing. The gene ontology analysis was performed with the r package "clusterprofiler" and r version 4.1.3. Data analyses on fastq files were conducted with CLC Genomics Workbench (version 22.0.2, QIAGEN, Venlo. NL). The commercial software package CLC bio started in 2005 and was acquired by QIAGEN in 2013. The complete analysis of the data presented in this manuscript was accomplished using the software version 22.0.2 of the CLC Genomics Workbench package (QIAGEN, Venlo. NL). Tools used within the CLC Workbench were the Differential Expression for RNA-Seq tool (version 2.7) and Gene Set Enrichment Test (version 1.2). After UMI (Unique Molecular Identifier) filtering, all remaining reads of all probes were adapter trimmed and quality trimmed (using the default parameters: bases below Q13 were trimmed from the end of the reads, ambiguous nucleotides maximal 2). Mapping was done against the *Mus musculus* (mm39; GRCm39.107) (July 20, 2022) genome sequence. After grouping of samples (for biological replicates each) according to their respective experimental condition, the statistical differential expression was determined using the Differential Expression for RNA-Seq tool (version 2.7). The resulting *p* values were corrected for multiple testing by FDR correction. A *p* value of ≤ 0.05 was considered significant. The Gene Set Enrichment Test (version 1.2) was done with default parameters and based on the GO term ‘biological process’ (*M. musculus*; December 16, 2021). Wald-test was used in the CLC Differential Expression for RNA-Seq tool (version 2.7) for the comparison of differences between pairs of groups (treated and untreated). ANOVA was used across groups for the heat map, based on the Likelihood ratio test. An FDR corrected *p* value of ≤ 0.05 (*P*adj) was considered significant. The comparison of each gene between the treated and untreated group was done by Wald-test. ANOVA was used across groups for the heat map. FDR was applied according to (Benjamini and Hochberg, 1995).

### Animals and ethics statement

All mice were kept under specific pathogen-free conditions and handled according to regulations defined by FELASA and the national animal welfare body GV-SOLAS (www.gv-solas.de). All animals were on C57BL/6N background and obtained from (Janvier, France). Mice were fed a standard laboratory diet and given autoclaved tap water *ad libitum*. They were kept in an air-conditioned room with controlled temperature (20–24 °C), humidity (45–65%), and day/night cycle (12 h light, 12 h dark). The experiments of this study were carried out according to the requirements of LANUV-NRW, Germany and approved by the LANUV-NRW (https://www.lanuv.nrw.de) with the approval number 81-02.04.2021.A064. GIL-6 or GIO-6 (20 μg) were injected intra-peritoneally in a total volume of 100 μl in PBS as vehicle.

### Protein expression, purification, and injection into mice

GIL-6, GIO-6, IC7, Hyper-CNTF, and Hyper-CNTF-IL-6R were produced and secreted as Fc-fusion by Expi293 cells (Thermo Fisher) and purified by Protein-A chromatography (HiTrap-MabSelect PrismA; Cytiva, catalog no. 17549852) according to the manufacturer’s manual. In order to induce cytokine signaling, mice were injected intraperitoneal (i.p.) with 20 μg GIL-6 or GIO-6 30 min before harvesting heart, liver, and spleen for to ensure distribution and signal transduction in the target cells followed by Western blot analysis.

### Immunoprecipitation

Protein A agarose beads (Roche Diagnostics Gmb; catalog no. 11134515001) were used for Immunoprecipitation (IP). Protein A agarose beads were incubated in 0.5 ml TBS-T (0.1% Tween-20) with 2 μg Hyper-CNTF with or without 1 μg recombinant human OSMR (catalog no. 4389-OR) with or without 1 μg biotinylated gp130 (catalog no. AVI11067-050), which were purchased from R&D Systems.

### Statistical analysis

Data are provided as arithmetic means ± SD using GraphPad Prism, Version 8. Statistically significant differences between two groups were determined with a Students’s *t* test, including Welch’s correction if indicated. Statistical analysis between several groups was determined using a two-way ANOVA, including Tukey or Dunnet’s correction. Significance was calculated as follows *p* > 0.05: n.s.; *p* < 0.05: ∗*p* < 0.01: ∗∗*p* < 0.001: ∗∗∗*p* < 0.0001: ∗∗∗∗.

### Data availability

The authors declare that the data supporting the findings of this study are available within the manuscript and from the authors on request. Gene expression data of 3′-RNA-Seq are available at NCBI Gene Expression Omnibus; accession code: GSE226064. The plasmid coding for GIL-6 and GIO-6 has been deposited at Addgene; Plasmid ID: 213389 and 213390, respectively, and will be provided upon request.

## Supporting information

This article contains supporting information.

## Conflict of interest

The authors declare the following financial interests/personal relationships which may be considered as potential competing interests:

J. S., P. R., and J. M. M. are inventors of GIL-6 and GIO-6 and hold patents for this molecule (EP22214005.5).
