# Supplementary material for: Engineered interleukin-6-derived cytokines recruit artificial receptor complexes and disclose CNTF signaling via the OSMR
Source: J Biol Chem. 2024 Apr 1;300(5):107251. doi: 10.1016/j.jbc.2024.107251 (PMC11039321; doi:10.1016/j.jbc.2024.107251)
Supplement: Supporting Information [file mmc1.pdf]

Supplement Figure 1

A

```
LIF                                               ILYYTAQGEPFPNNLDKLCGP
GIL-6      VPPGEDSKDVAAPHRQPLTSSERIDKQIRYILDGISALRILYYTAQGEPFPNNLDKLCGP 60
IL-6       VPPGEDSKDVAAPHRQPLTSSERIDKQIRYILDGISALRKETCNKSNM---CESSKEALA 57
           ***************************************    . ..     : .* .

LIF        N                                          TRDQKILNPSALSLHSK
GIL-6      NNNLNLPKMAEKDGCFQSGFNEETCLVKIITGLLEFEVYLEYLTRDQKILNPSALSLHSK 120
IL-6       ENNLNLPKMAEKDGCFQSGFNEETCLVKIITGLLEFEVYLEYLQNRFE-------SSEEQ 110
           :****************************************** .  :       * ..:

LIF        L                                       TSGKDVFQKKKLGCQLL
GIL-6      LRAVQMSTKVLIQFLQKKAKNLDAITTPDPTTNASLLTKLTSGKDVFQKKKLGCQLLRSF 180
IL-6       ARAVQMSTKVLIQFLQKKAKNLDAITTPDPTTNASLLTKLQAQNQWLQ-DMTTHLILRSF 169
           *************************************** : :: :* .     :****

LIF
GIL-6      KEFLQSSLRALRQM  194
IL-6       KEFLQSSLRALRQM  183
           **************
```

B

```
OSM                                               DPYIRIQGLDVPKLREHCRER
GIO-6      VPPGEDSKDVAAPHRQPLTSSERIDKQIRYILDGISALRDPYIRIQGLDVPKLREHCRER 60
IL-6       VPPGEDSKDVAAPHRQPLTSSERIDKQIRYILDGISALRKETCNKSNM--CESSKEALAE 58
           ***************************************.   . ..:   :  :..  .

OSM                                                  EQRLPKAQDLERSGLNIE
GIO-6      NNLNLPKMAEKDGCFQSGFNEETCLVKIITGLLEFEVYLEYLEQRLPKAQDLERSGLNIE 120
IL-6       NNLNLPKMAEKDGCFQSGFNEETCLVKIITGLLEFEVYLEYLQNRFESSE---------- 108
           ******************************************::*: .::

OSM        DLEKL                                       TPTPASDAFQRKLEGC
GIO-6      DLEKLRAVQMSTKVLIQFLQKKAKNLDAITTPDPTTNASLLTKLTPTPASDAFQRKLEGC 180
IL-6       --EQARAVQMSTKVLIQFLQKKAKNLDAITTPDPTTNASLLTKLQA---QNQWLQDMTTH 163
             *: ***************************************     .: : :.:

OSM        RFL
GIO-6      RFLRSFKEFLQSSLRALRQM 200
IL-6       LILRSFKEFLQSSLRALRQM 183
            :******************
```

C

```
GIO-6      VPPGEDSKDVAAPHRQPLTSSERIDKQIRYILDGISALRDPYIRIQGLDVPK-LREHCRE 59
IC7        VPPGEDSKDVAAPHRQPLTSSERIDKQIRYILDGISALRESYVKHQGLNKNINLDSADG- 59
GIL-6      VPPGEDSKDVAAPHRQPLTSSERIDKQIRYILDGISALRILYYTAQGEPFPNNLDKLCGP 60
           ***************************************  *   **      * .

GIO-6      RNNLNLPKMAEKDGCFQSGFNEETCLVKIITGLLEFEVYLEYLEQRLPKAQDLERSGLNI 119
IC7        MNNLNLPKMAEKDGCFQSGFNEETCLVKIITGLLEFEVYLEYLLEDQQV----HFTPTEG 115
GIL-6      NNNLNLPKMAEKDGCFQSGFNEETCLVKIITGLLEFEVYLEYLTRDQKI-----LNPSAL 115
            ****************************************** .          .

GIO-6      EDLEKLRAVQMSTKVLIQFLQKKAKNLDAITTPDPTTNASLLTKLTPTPASDAFQRKLEG 179
IC7        DFHQAIRAVQMSTKVLIQFLQKKAKNLDAITTPDPTTNASLLTKLGD--G-GLFEKKLWG 172
GIL-6      SLHSKLRAVQMSTKVLIQFLQKKAKNLDAITTPDPTTNASLLTKLTS--GKDVFQKKKLG 173
           .  . :***************************************    . . *::*  *

GIO-6      CRFLRSFKEFLQSSLRALRQM 200
IC7        LKVLRSFKEFLQSSLRALRQM 193
GIL-6      CQLLRSFKEFLQSSLRALRQM 194
            :.******************
```

Supplemental Figure 1. (A) Amino acid sequence alignment of human LIF, GIL-6 and human IL-6. excluding signal peptide **(B)** Amino acid sequence alignment of human OSM, GIL-6 and human IL-6. **(C)** Amino acid sequence alignment of the cytokimera GIO-6, IC7 and GIL-6 excluding signal peptide.

Supplement Figure 2

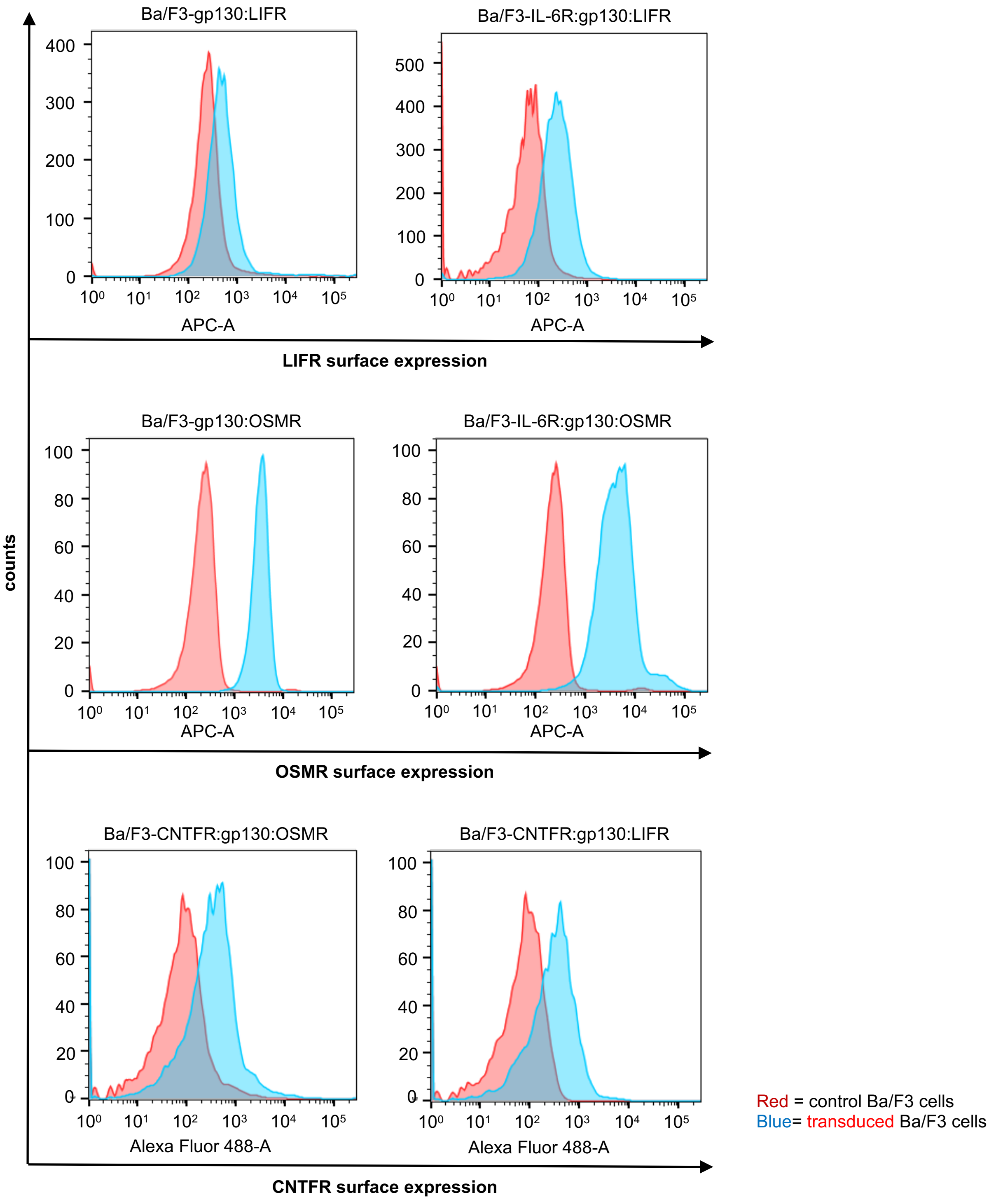

Supplemental Figure 2. Receptor cell surface expression of Ba/F3-gp130:LIFR or Ba/F3-IL-6R:gp130:LIFR cells transduced with human LIFR (blue shades), Ba/F3-gp130:OSMR or Ba/F3-IL-6R:gp130:OSMR cells transduced with human OSMR (blue shades) and Ba/F3-CNTFR:gp130:OSMR or Ba/F3-CNTFR:gp130:LIFR cells transduced with human CNTFR (blue shades) compared with non-transduced control cells (red shades) by flow cytometry. Expression was demonstrated via receptor-specific antibodies.

Supplement Table 1

Supplemental Table 1. Significant regulated genes by natural or synthetic cytokines. Determined by 3'-RNA-Seq Analyses of Ba/F3-IL-6R:gp130:LIFR cells. **(A)** All genes that are overexpressed by natural or synthetic cytokines are listed. Filter: $p<0.05$ including false discovery rate correction; Fold change >=1.5. **(B)** All genes that are suppressed by natural or synthetic cytokines are listed. Filter: $p<0.05$ including false discovery rate correction; Fold change <=1.5.

A

FC>1.5; FDR<0.05

Name	Total	Elements
GIL-6 GIO-6 IC7 IL-6 LIF	44	*Btg2 Egr2 Junb Spry2 Gcc1 Ier2 Gpr146 Il17ra Osm Socs1 Notch1 Fos Myd88 Pim1 Socs3 Id2 Arid5a Il4ra Cish Hk2 Il10 Chd7 Sbno2 Nfkbiz Id1 Adora3 Mycn Tiparp Il6 Serpina3g Cebpd Jun Bcar3 Zfp36l1 Csrnp1 Nfil3 Ssh1 Ier3 Cdkn2d Gadd45g Irf1 Ier5 Gpr171 Zfp36*
GIL-6 GIO-6 IC7 LIF	1	*Gpr65*
GIL-6 IC7 IL-6 LIF	6	*Dusp1 Irf2bpl P2ry13 Bcl6 Tagap Phlda1*
IC7 IL-6 LIF	3	*Fosl2 Cgas Midn*
GIL-6 IL-6 LIF	2	*Cldn12 Map3k8*
GIO-6 IL-6 LIF	2	*Mex3a Selp*
IC7 LIF	3	*Tnf Atg4d Zc3h12a*
GIO-6 IL-6	1	*Tnfaip2*
IL-6 LIF	10	*Smad7 Bhlhe40 Pde12 Dusp5 Bnip5 Nr4a2 Tasl Bmf Stk17b Cxcr2*
IC7	1	***Prickle1***
GIL-6	1	*Vmn1r47*
GIO-6	1	***Ifitm5***
IL-6	3	*Kctd11 Tob1 Il1b*
LIF	17	*Serpinb1a Metrnl Kdm6b Dusp2 Ddah2 Casp4 Etv3 Prdm1 Nupr1 Slc41a1 Gadd45b Batf Hbegf Pim2 Vmp1 Lpar6 Slc25a30*

B

FC < -1.5; FDR < 0.05

Name	Total	Elements
IC7	3	*Gse1 Dynlt1a Atl1*
IL-6	2	*E130218I03Rik Kif5a*
LIF	2	*Zfp773 Zfp553*

Supplement Figure 3

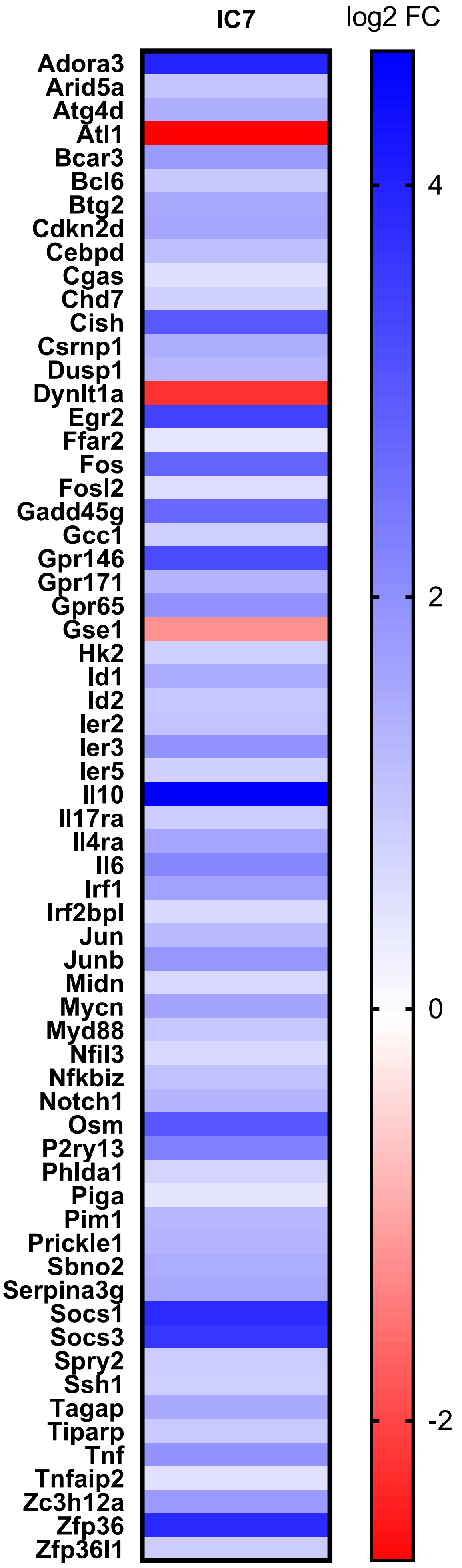

Supplemental Figure 3. Heat map shows 64 genes that are significantly regulated by IC7 vs untreated Ba/F3 cells expressing gp130, LIFR and IL-6R. Filter: $p<0.05$ including false discovery rate correction. Scale bar shows log(2) fold change of upregulated (blue), not regulated (white) and downregulated (red) genes.

Supplement Figure 4

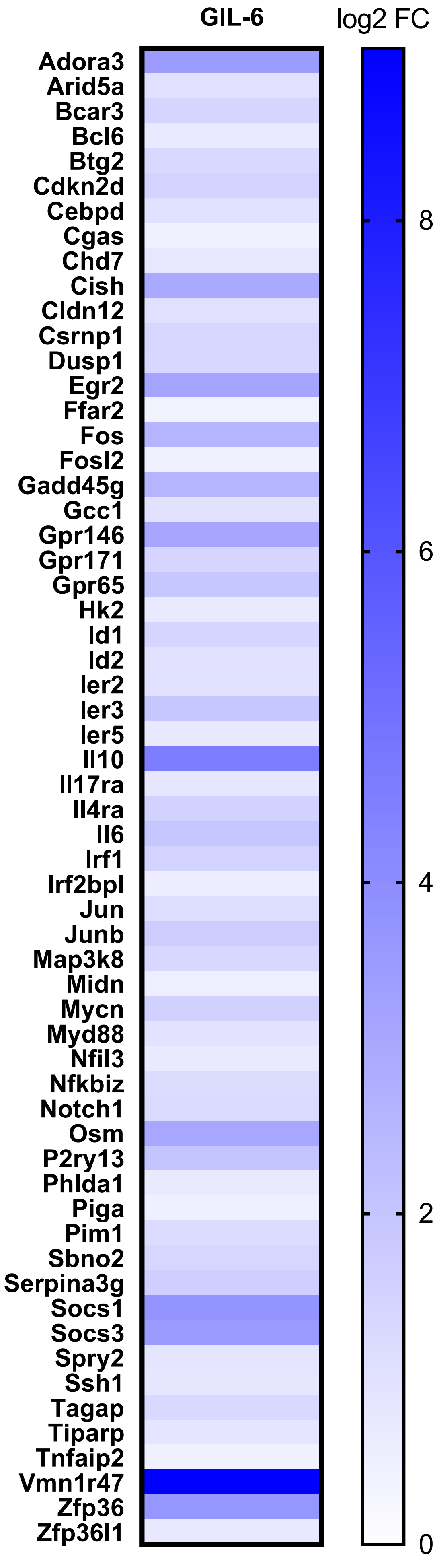

Supplemental Figure 4. Heat map shows 60 genes that are significantly regulated by GIL-6 vs untreated Ba/F3 cells expressing gp130, LIFR and IL-6R. Filter: $p<0.05$ including false discovery rate correction. Scale bar shows log(2) fold change of upregulated (blue) and not regulated (white) genes.

Supplement Figure 5

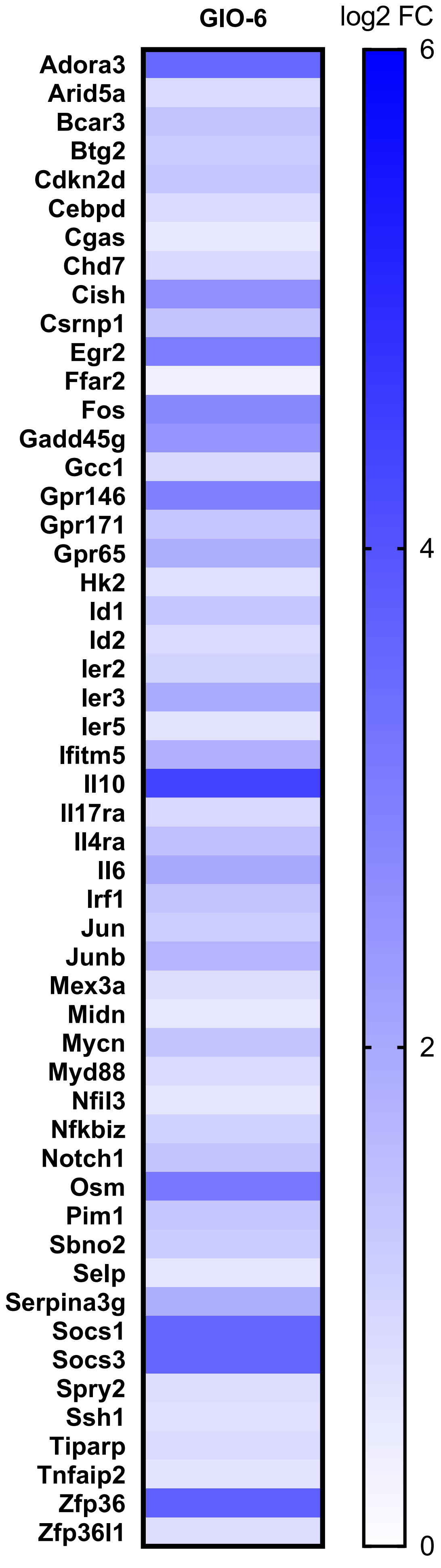

Supplemental Figure 5. Heat map shows 52 genes that are significantly regulated by GIO-6 vs untreated Ba/F3 cells expressing gp130, LIFR and IL-6R. Filter: $p<0.05$ including false discovery rate correction. Scale bar shows log(2) fold change of upregulated (blue) and not regulated (white) genes.

Supplement Figure 6

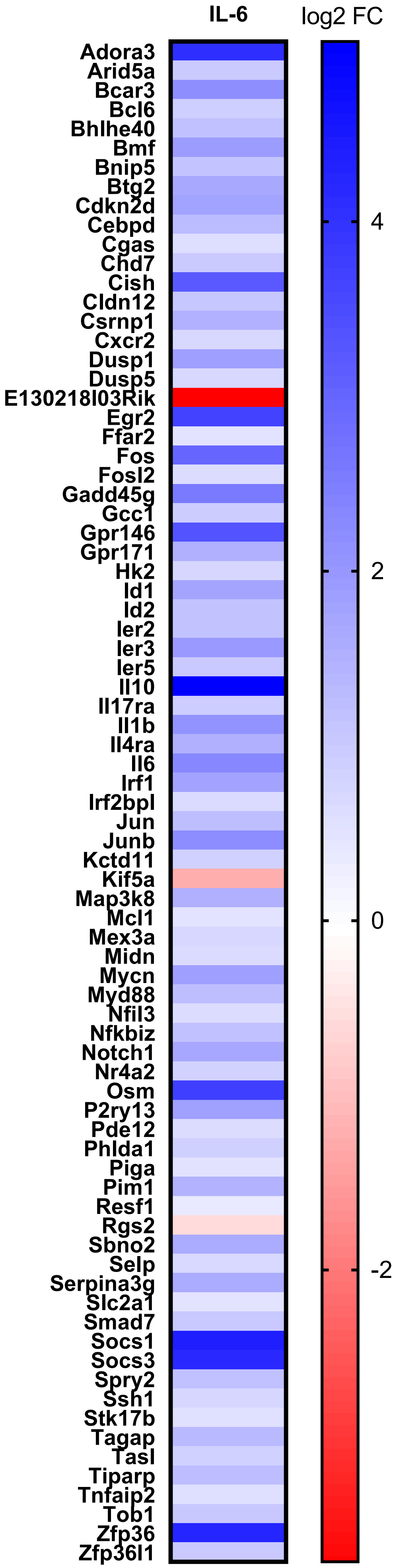

Supplemental Figure 6. Heat map shows 79 genes that are significantly regulated by IL-6 vs untreated Ba/F3 cells expressing gp130, LIFR and IL-6R. Filter: *p*<0.05 including false discovery rate correction. Scale bar shows log(2) fold change of upregulated (blue), not regulated (white) and downregulated (red) genes.

Supplement Figure 7

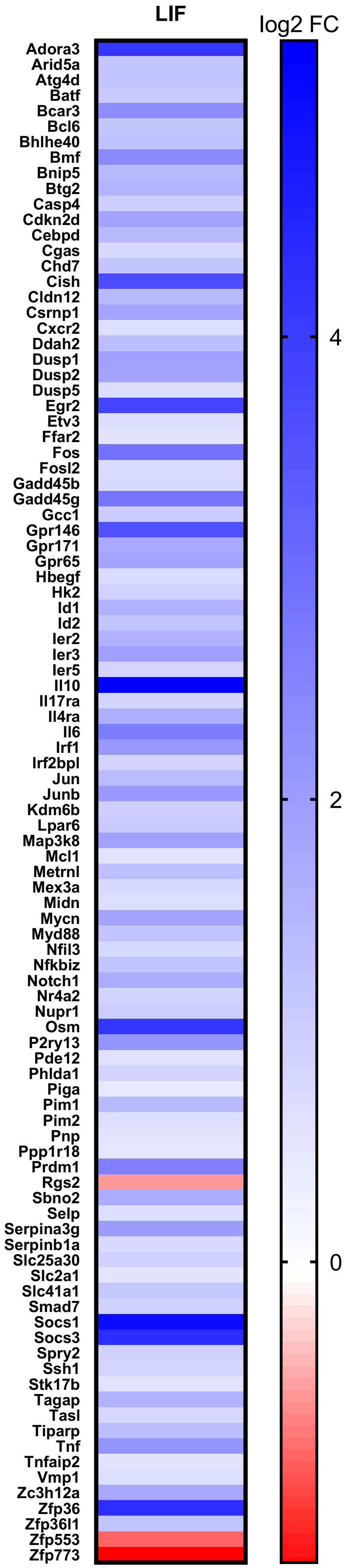

Supplemental Figure 7. Heat map shows 98 genes that are significantly regulated by LIF vs untreated Ba/F3 cells expressing gp130, LIFR and IL-6R. Filter: $p<0.05$ including false discovery rate correction. Scale bar shows log(2) fold change of upregulated (blue), not regulated (white) and downregulated (red) genes.
